# FACTORS ASSOCIATED WITH OLDER ADULTS’ READINESS FOR DISCHARGE FROM POST ACUTE CARE TO HOME

**DOI:** 10.1093/geroni/igad104.3342

**Published:** 2023-12-21

**Authors:** Susan Barnason

**Affiliations:** University of Nebraska Medical Center, Lincoln, Nebraska, United States

## Abstract

Following acute hospitalization, older adults are often transferred to post-acute care (PAC) for rehabilitation and nursing care. Most PAC admissions are comprised of adults with multimorbidity (>2 chronic diseases). After PAC discharge many patients are rehospitalized within 30 days. The goal of this study was to evaluate factors associated with older adult’s readiness for PAC discharge to home. A prospective one-arm descriptive correlation study was conducted. Older adults (N=19) discharged from PAC to home participated. Subjects were recruited over a 3-month period from a large midwestern facility. Participants completed the Patient Readiness for Hospital Discharge -Short Form (PR RHDS-SF) and Patient Activation Measure (PAM). Medication burden, measured by the medication regimen complexity index (MRCI) calculated from discharge medication record. Participants included 12 females and 5 males, mean age of 73 (SD 8.2)) years. The PT RHDS -SF mean score was 49.3 (SD 16.8). The PAM mean score was 54.7 (SD 7.4). Medication burden was high, mean MRCI was 31.5 (SD 11.6). There was a significant correlation [r (17) .48, p=.04] between total PT RHDS-SF score and patient activation levels. Preparing PAC patients to self-manage their multimorbid conditions may enhance their self-confidence with transition from PAC to home. Evaluating self-management preparedness of older adults prior to PAC discharge is vastly understood. Research is needed to prepare multimorbid older adult patients transitioning from PAC to home focused on perceived readiness and self-management confidence.

